# The Medical Student’s Vade Mecum, Containing Examinations upon Anatomy, Chemistry, Materia Medica, Surgery, Practice of Medicine, Obstetrics and Poisons

**Published:** 1845-02

**Authors:** 


*The Medical Student's Vade Mecum, containing Examinations upon Anatomy, Chemistry, Materia Medica, Surgery, Practice of Medicine, Obstetrics and Poisons. Adapted to the use of Medical Students generally.* By George Mendenhall, M. D., &c. One volume, 12mo. pp. 328. Cincinnati: Jacob Ernst. 1844.

"The object of this little volume," the author tells us, "is to provide the Student of Medicine with a short and succinct view of the most important facts and principles which engage his attention during his Academic studies, in order that he may refresh, and fix more firmly upon his memory, what he has read and heard; as well as to enable him to arrange properly his knowledge, so as to make a right use of it." This publication, we doubt not, is as well adapted to the objects set forth in the above extract as any other of its size: indeed, we regard it as possessing decided advantages over most of them; yet, after all, we cannot but look upon all such works as at best "poor helps." The industrious student will seek, in works of greater extent on the various branches of science, fuller information on all the topics he designs to master, and will find in his abridged notes of what he reads, better means of fixing it in his memory, so as to render it available for all necessary occasions. Too many, it is to be feared, rely upon this kind of assistance to the neglect of legitimate study, and as a substitute for proper office training. The mode of conveying instruction by questions and answers may do very well, provided the answers are sufficiently comprehensive and explicit; but that can hardly be in a small volume devoted to all the branches of medical science. Questions, which are not prompted by the deficiencies of the student, and brief answers, which are not specially adapted to supply those deficiencies, by varied phrase and illustration, it appears to us, are of doubtful utility. Abstract truths may be conveyed in that way, but not the philosophy of science.

